# Development and validation of a weight-loss predictor to assist weight loss management

**DOI:** 10.1038/s41598-023-47930-y

**Published:** 2023-11-24

**Authors:** Alexander Biehl, Mikko S. Venäläinen, Laura U. Suojanen, Sakris Kupila, Aila J. Ahola, Kirsi H. Pietiläinen, Laura L. Elo

**Affiliations:** 1 Turku Bioscience Centre, University of Turku and Åbo Akademi University, Tykistökatu 6 A, 20520 Turku, Finland; 2 Obesity Research Unit, Research Program for Clinical and Molecular Metabolism, University of Helsinki, Helsinki, Finland; 3 Folkhälsan Institute of Genetics, Folkhälsan Research Center, Helsinki, Finland; 4 Department of Nephrology, University of Helsinki and Helsinki University Hospital, Helsinki, Finland; 5 Obesity Center, Endocrinology, Abdominal Center, University of Helsinki and Helsinki University Central Hospital, Helsinki, Finland; 6 Institute of Biomedicine, University of Turku, Turku, Finland

**Keywords:** Machine learning, Public health, Weight management

## Abstract

This study aims to develop and validate a modeling framework to predict long-term weight change on the basis of self-reported weight data. The aim is to enable focusing resources of health systems on individuals that are at risk of not achieving their goals in weight loss interventions, which would help both health professionals and the individuals in weight loss management. The weight loss prediction models were built on 327 participants, aged 21–78, from a Finnish weight coaching cohort, with at least 9 months of self-reported follow-up weight data during weight loss intervention. With these data, we used six machine learning methods to predict weight loss after 9 months and selected the best performing models for implementation as modeling framework. We trained the models to predict either three classes of weight change (weight loss, insufficient weight loss, weight gain) or five classes (high/moderate/insufficient weight loss, high/low weight gain). Finally, the prediction accuracy was validated with an independent cohort of overweight UK adults (n = 184). Of the six tested modeling approaches, logistic regression performed the best. Most three-class prediction models achieved prediction accuracy of > 50% already with half a month of data and up to 97% with 8 months. The five-class prediction models achieved accuracies from 39% (0.5 months) to 89% (8 months). Our approach provides an accurate prediction method for long-term weight loss, with potential for easier and more efficient management of weight loss interventions in the future. A web application is available: https://elolab.shinyapps.io/WeightChangePredictor/.

The trial is registered at clinicaltrials.gov/ct2/show/NCT04019249 (Clinical Trials Identifier NCT04019249), first posted on 15/07/2019.

## Introduction

During the last few decades, the prevalence of overweight and obesity has increased worldwide. In 2016, 1.9 billion adults (39%) had overweight, of which 650 million (13%) had obesity^1^. Both conditions are associated with a number of comorbidities: type 2 diabetes mellitus, coronary heart disease, increased incidence of certain forms of cancer, respiratory complications, osteoarthritis, hypertension and more^2, 3^. Similar risks exist with obesity during childhood, which seems to have an effect on health risks in adulthood, even after successful loss of the extra weight during adulthood^4^. These overweight and obesity related health issues place a financial burden on the health systems today, estimated as combined lifetime costs for example for boys with obesity to approximately € 150,000^5^. In 2008, medical costs connected with overweight or obesity caused medical expenses of approximately 114 billion in the US alone^6^.

It is known that even a modest weight loss of 5–10% significantly reduces the risk of cardiovascular disease in overweight and the risk of type 2 diabetes in obesity and that a greater weight loss leads to even greater risk reductions^7^. Despite the benefits of weight loss, only about 60% of participants adhere to their weight loss program for the planned time^8^. Successfully lost weight is also often regained. On average about 55% of the lost weight is regained after 2 years and about 80% after 5 years^9^. However, there are simple procedures, such as reinforcement interventions during the weight loss phase, which have been shown to lead to significantly better weight loss success^10^. This suggests that individuals, who are at risk of not achieving their weight loss goals, could be helped with additional interventions by healthcare professionals.

To aid healthcare professionals with planning and monitoring weight loss interventions, different computational approaches have been applied to predict expected weight loss. The most popular approaches for weight loss predictions are based on the balance between energy intake and expenditure^11^. Despite their popularity, the downside of the energy-based weight loss models complicating their use is that individuals tend to estimate the energy intake incorrectly^12, 13^. As an alternative approach, it has been reported that prediction accuracies of about 80% could be achieved for short time frame binary prediction (e.g., if a 10% percentage weight loss goal would be achieved or not) by using just the baseline information such as body mass index (BMI), age and initial weight, but with high false negative rates^14^. It has also been demonstrated that early weight loss results, for example at 1 month, can be important predictors for weight loss success at 12 months^15^. However, to the best of our knowledge, there are currently no methods taking advantage of weight loss results in a continuous manner that would be simple to apply and do not rely on uncertain estimates of energy balance.

To address the need, the aim of this work was to apply machine learning methods to develop and test a modeling framework for predicting expected weight loss based on self-reported weight data in a continuous manner and develop an application which helps monitoring weight loss success during interventions. We hypothesize that by taking longitudinal weight data into account, weight loss predictions can be achieved with good accuracy.

## Materials and methods

### Training cohort

In order to develop a modeling framework for predicting expected weight loss based on longitudinal weight data, we used data from a large real-life cohort (n = 1453) of participants in a web-based program, healthy weight coaching (HWC)^16^. In short, the dataset contains weight data collected during a 12-month interactive web-based intervention for weight management in obesity. It includes submitting weight and diet logs by the participants using a web application, through which they get automated feedback for the submissions, anonymous contact to other participants and support by an assigned personal coach. Each participant provided written informed consent. The study was approved by the Coordinating Ethics Committee of the Helsinki University Hospital (Reference Number 327/13/03/00/2015). All methods were carried out in accordance with relevant guidelines and regulations. All participants were referred by a licensed physician and needed to fulfill the criteria of age of 18 years or older, BMI of 25 kg/m^2^ or higher, access to computer/smartphone and willingness to participate. In the present study, we excluded the individuals that fulfilled any of the following criteria, to remove outlier entries introduced by individuals and influence of weight loss medication:

1. known prescription of weight-loss medicine
2. less than two weight entries during the 9 months
3. more than 1.5 kg/day weight change at any point
4. weight data available for less than 270 days
5. height of 100 cm or less

This left us with data from 327 individuals for training the prediction models. The average number of weight data points for the selected individuals was 36 ± 19 (range: 3–149). The selection process is also shown graphically in the Supplementary Figure S1. Most data was removed due to (4), as this cohort is continously recruiting, many participants were not enrolled for 270 days yet.

### Clustering for output variable

We trained our models for two prediction scenarios: (1) Overall three-class weight loss result at 9 months (weight gain, insufficient weight loss, weight loss) and (2) A more refined five-class prediction of weight loss result at 9 months (high/low weight gain, high/moderate/insufficient weight loss), as explained in more detail below. Due to continuous online recruitment of new participants, not all participants had reached the end of the program (12-month time point) at the time of the data lock. Therefore, we selected the 9-month time point (one month defined as 30 days) to increase the number of individuals reaching the final time point of the current analyses (327 participants) compared to 12 months (173 participants).

To define the five weight change classes (high/moderate/insufficient weight gain, high/low weight loss), we applied three different versions of agglomerative hierarchical clustering to the HWC weight change data. The distance function used was dynamic time warping (DTW), which is able to detect similarities between time series data^17, 18^. Our aim by using DTW as distance function was to be more focused on the shape of the time series, not only the final weight change value. For example, an individual with high initial weight loss and then reaching a plateau should have a small distance to an individual with slow weight loss, but then a plateau at the same level as the first individual. The first hierarchical clustering was implemented using the *R* (v 3.5.3)^19^ package *dtwclust*^20^. The other two hierarchical clusterings were obtained using the *Python* (v 3.6.9)^21^ package *sklearn* (v 0.22.1)^22^ with the DTW implementation of either the *dtaidistance* (v 1.1.4)^23^ or *cdtw* (v. 0.0.1)^24^ package. We computed the mean weight changes for each cluster by only considering individuals, where all three implementations agreed on the cluster (59.5%). Finally, these mean values were refined by applying the k-means algorithm to the entire HWC dataset, using the means from the first step as initial centroids and DTW as distance function. To determine the three weight change classes for the simpler prediction scenario, we combined the high/moderate weight loss classes and the high/low weight gain class into one class, respectively.

### Input variables

Each model was trained using weight change data from nine different time frames, all starting at baseline and spanning up to half, 1, 2, 3, 4, 5, 6, 7 or 8 months. The weight changes were calculated in percent in relation to the baseline weight. Data for the days between the weight entries were linearly interpolated. When applied to new individuals, the predictions were done using the model trained for the time frame closest to the available weight change datum of that individual; for instance, if three and a half month’s weight change datum for an individual was available, the model trained for the 4-month time frame was used for prediction.

**Figure 1:**
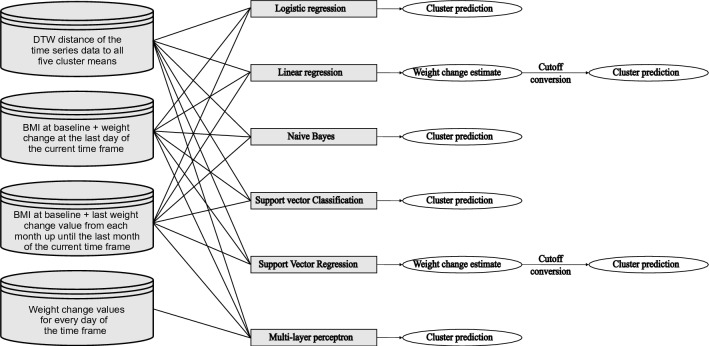
Input variable to output variable flow with the six different machine learning models used.

For training the models with each method, we tested three different types of input data to identify the best approach for each method: (1) BMI at baseline together with the weight change at the last day of the respective time frame, (2) BMI at baseline together with the last weight change value from each time frame up until the current one, and (3) DTW distances between the observed series and the cluster means. With multi-layer perceptron, we also tested the daily weight change values as additional input data type. We then selected the input data that achieved the best averaged cross-validation prediction accuracy (Table 1). The fold assignment for the fivefold cross-validation was done by randomly sampling an equal number of observations from each prediction cluster. The flow from input data to the outcome is illustrated in Fig. 1.

**Table 1:** Prediction models with their type and description of input data used for prediction. 1 Dynamic time warping.

Model	Type	Input data
Logistic regression	Classification	DTW^1^ distance of the time series data to all five cluster means
Linear regression	Regression	DTW distance of the time series data to all five cluster means
Naive Bayes	Classification	BMI at baseline, weight change at the last day of the respective time frame
Support vector classification	Classification	DTW distance of the time series data to all five cluster means
Support vector regression	Regression	BMI at baseline, weight change at the last day of the respective time frame
Multi-layer perceptron	Classification	BMI at baseline, weight change at the last day of the respective time frame

### Model development

For predicting the cluster-based 9-month weight-loss results, we considered the following general statistical model

$$y = f\left( {x_{t} } \right) + \epsilon _{t}$$

where $y$ is the observed weight loss outcome (either the five- or three-class weight loss result defined in section “Clustering for output variable” or the 9-month weight loss in percentages), $f$ is an unknown function relating an input vector ${x}_{t}$ consisting of data available at time point $t$ (defined in section “Input variables”) to $y$, and ${\epsilon }_{t}$ is a random error term which is independent of ${x}_{t}$ and has mean zero and constant variance. To estimate $f$, we applied six supervised machine learning methods: logistic regression, linear regression, naive Bayes classification, support vector classification, support vector regression, and multi-layer perceptron. In all approaches, our primary aim was to test the method for predicting the correct weight loss cluster instead of using the modelling coefficients for statistical inference and hence we did not strictly require, e.g., the independence of the input variables. All models were trained on the HWC dataset using fivefold cross-validation, and later validated with the independent Oxford Food and Activity Behaviours (OxFAB) cohort study dataset^25^. Details for all six models can be found in the Supplementary Text.

The regression-type models predicted the weight loss in percentages, which afterwards were converted into the classes using cutoff values in weight change percentages. We did this to be able to directly compare the results to the classification models. To compute the cutoff values, we sorted the observed weight change values after 9 months of each individual from high to low and selected the value as cutoff that minimized the number of classification errors. If there were several values with the same error, we selected the largest cutoff value.

### Applying the models to real-world data

Predictions for an individual were calculated only, when the following criteria were fulfilled for the current prediction time frame:

1. at least two weight entries of the individual within the current prediction time frame
2. the last weight entry within 30 days of the end of the current prediction time frame

This ensured that each prediction was only tested for those cases who would also be computed in clinical use.

### Computational software

Data analysis and computational modeling was done in *Python* (v 3.6.9). For the machine learning models, we used the package *sklearn* (v 0.22.1). The *dtaidistance* package (v 1.1.4) was used for the DTW implementation.

### Independent validation

All prediction models were validated with independent data from the previously published OxFAB cohort study (n = 1265). In addition to the baseline data, the OxFAB dataset also contains self-reported weight data up to 1 year of follow-up. Medication data were not available for the OxFAB dataset and, therefore, exclusion criterion (1) used for the HWC dataset was omitted here, while the remaining exclusion criteria were used to exclude the outliers in the validation dataset. We also used one additional criterion: at least one weight entry within the first 270 days.

This left us with data from 184 individuals for validating the prediction models. The average number of weight data points for the selected individuals was 16 ± 16 (range: 3–162). The selection process is also shown graphically in the Supplementary Figure S1. For the validation datasets, data were available as follows: n = 46 (0.5 months), n = 60 (1 month), n = 83 (2 months), n = 126 (3 months), n = 104 (4 months), n = 83 (5 months), n = 71 (6 months), n = 66 (7 months), and n = 62 (8 months). Again, missing weight data were linearly interpolated using adjacent weight entries. To match the situation in clinical use, we interpolated within the prediction time frame until the last available weight entry and then extrapolated the weight change after the last entry until the end of the prediction time frame using the average change of weight per day between the baseline and the last weight entry within the prediction time frame.

The truth, against which the prediction was compared, was determined by computing the DTW distance between the weight change data of each individual and the means of the five clusters derived from the training dataset. Each individual was assigned to the cluster with the smallest distance, similarly as in the training data. Accuracy was defined as the percentage of predictions that matched the truth.

To compare the multi-class accuracies against a random model for both the three- and five-class prediction models, we computed the percentage of participants in each class in the training data and randomly selected one class according to these probabilities. To compute a good estimate of the average accuracy, this was repeated 10,000 times on the validation dataset and the results were averaged.

### Prior present

Parts of the study were previously presented in abstract form at the Applied Bioinformatics in Life Sciences (3rd edition) conference 13–14 February 2020 in Leuven, Belgium.

## Results

### Clustering to define weight loss results

**Figure 2:**
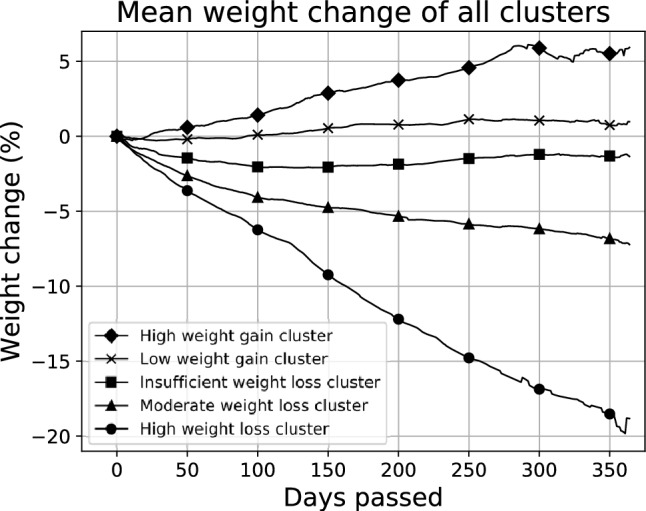
Mean weight change of the five clusters obtained as a result of applying the k-means algorithm on the Healthy Weight Coaching dataset.

Figure 2 shows the mean weight change of the five clusters obtained as a result of applying the k-means algorithm to the self-reported weight data from the HWC cohort. The dataset was split in two clusters with high/moderate weight loss, one cluster with insufficient weight loss, and two clusters with high/low weight gain. The weight loss cutoff values were determined as − 9.2%, − 2.4%, − 0.1% and 2.2% for the five-class prediction and − 2.4% and − 0.1% for the three-class overall weight loss prediction.

The high weight loss cluster was characterized by a majority of the individuals (88%) reaching a weight loss of > 10% whereas in the moderate weight loss cluster, a majority of the individuals (71%) reached a weight loss of 5–10%. In the insufficient weight loss cluster, the individuals did not lose substantial weight (weight loss of 0–3%, 63%). The individuals in the remaining low and high weight gain clusters gained 0–3% (78%) and > 3% (81%), respectively.

### Prediction accuracy

In the fivefold cross-validation in the HWC cohort, logistic regression performed best (three-class accuracy from 54% at 0.5 months to 85% at 8 months, five-class from 35% at 0.5 months to 79% at 8 months), followed by support vector classification (three-class from 46 to 85%, five-class from 33 to 75%). The overall lowest accuracies were obtained with linear regression (three-class from 44 to 77%, five-class from 38 to 68%) and support vector regression (three-class from 39 to 78%, five-class from 36 to 72%). All models improved with longer follow-up data. As expected, for the more refined five-class prediction, the accuracies of all the models were somewhat lower than for the more general three-class prediction. The complete cross-validation results are provided in Supplementary Table S1.

**Figure 3:**
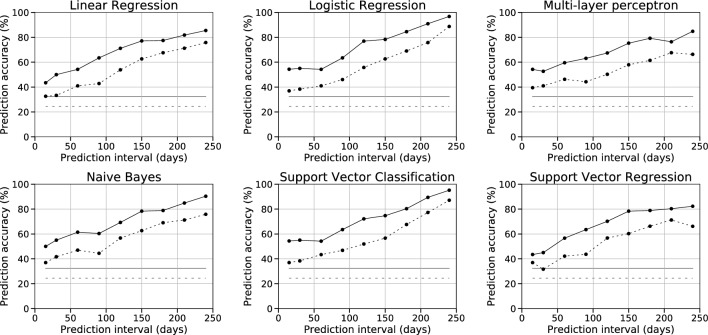
Prediction accuracies of the six machine learning methods in the independent validation dataset. The solid line shows the prediction accuracy for the three-class overall weight loss prediction model (weight gain, insufficient weight loss, weight loss), the dotted line shows the prediction accuracy for the more refined five-class prediction model (high/low weight gain, high/moderate/insufficient weight loss). The grey lines show the prediction accuracy for the random model.

Next, we tested the models built using the HWC cohort in the independent OxFAB validation cohort. In line with the cross-validation results, for the three-class overall weight loss prediction, most of the models reached prediction accuracy of ~ 50% already at half a month, reaching up to ~ 97% after 8 months (Fig. 3, solid lines). Again, logistic regression and support vector classification performed generally best. For the five-class prediction, the accuracies of logistic regression and support vector classification ranged from 37% at half a month to close to 90% at 8 months (Fig. 3, dotted lines). For comparison, the random prediction model accuracies were 32% for the three-class prediction and 24% for the five-class prediction. The complete results can be found in the Supplementary Table S2. Additionally, detailed class-based performance measures (accuracy, sensitivity, specificity, precision, and F1-score) as well as confusion matrices for all models except multi-layer perceptron can be found in Supplementary Table S3. The coefficients for the best-performing logistic regression models can be found in Supplementary File S1.

Finally, to demonstrate the ability of our model to predict non-respondence to intervention, we calculated the accuracy of a binary prediction for the best-performing logistic regression model by combining the weight gain and insufficient weight loss clusters of the three-class prediction model. Already at 2 and 3 months, we achieved accuracies of 64% and 73%, respectively.

### Prediction model for the web application

To demonstrate how the best-performing models could be used in practice, we developed a simple-to-use online tool on *R* statistical computing environment (v 3.5.3) using the *R* package shiny (v 1.4.0)^26^. Based on the evaluation of the modeling approaches, we selected logistic regression both as the prediction model for the overall weight loss result and for predicting the more detailed weight loss result. Support vector classification performed similarly as well, but was overall slightly outperformed by logistic regression.

**Figure 4:**
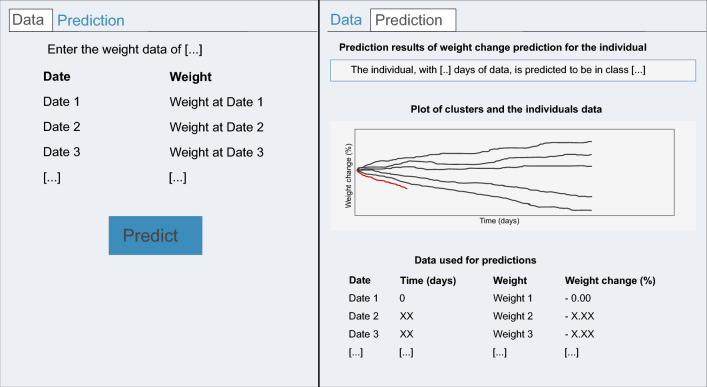
Illustration of the web application. Left side: input form for the individual weight data. Right side: summary of the results, including the prediction, visualization of the weight change profile in comparison to the five weight change clusters and the weight data in table form.

On the index page of the web application, a user can input weight data for an individual or use the given example data (Fig. 4, left side). To make a prediction, the tool processes the weight data in the same way as the OxFAB data to compute the weight change data, to extrapolate the data until the end of the time frame and to calculate the DTW distance to the means of the five weight-change clusters. If the data span up to 4 months, we applied the respective three-class logistic regression model, if they span longer than 4 months, we applied both the respective three-class logistic regression and five-class logistic regression model. The weight change profile is then displayed together with the prediction result in the prediction tab (Fig. 4, right side). The developed tool is freely available at the Shinyapps.io (RStudio Inc.) service-platform: https://elolab.shinyapps.io/WeightChangePredictor/

## Discussion

As overweight, obesity and their co-morbidities increase in numbers worldwide, weight loss interventions become more important in health care. Weight loss prediction could be a helpful tool for healthcare professionals and participants in weight loss interventions. In this study we evaluated several machine learning models and their ability to predict long-term weight loss using baseline- and longitudinal weight data self-reported by individuals. Comparing accuracies of all models with the corresponding random model accuracy, we saw that all models, even for the half a month time frame, had considerably better prediction accuracies than the random prediction. We selected the best models to implement a prediction framework for application in healthcare.

So far, the dominant type of prediction models for long-term weight loss are based on baseline data. With baseline data, the previously published model reported accuracy of 80% with cross-validation, when predicting binary outcomes (weight loss greater/smaller than 10%) after 8 weeks^14^. We can compare that to our prediction model trained using 7 months of data, which also predicts an 8-week period (7 months to 9 months). Our three-class model achieved 91% prediction accuracy and even the more refined five-class model achieved accuracy of 76% in the independent validation data. Validation with independent data also eliminates any risk of overfitting our models to the training data. Energy uptake and expenditure-based models are also used, but mostly for short-term prediction. They can model weight loss very well for the time frame of 1 week^27^ but suffer when the energy data are estimated by the users themselves, as they frequently estimate incorrect values^12, 13^. Our model provided long-term prediction far exceeding time frames of 1 week, while relying only on weight data, which is very simple for users to measure and to report.

For weight loss interventions, it is useful to be able to tell as early as possible, if an individual is responding or not responding. During the first months, the approach of binary prediction allows this to a higher degree, as shown for logistic regression for time frames 2 and 3 months. This is similar performance as with earlier prediction models^28^, which would allow additional intervention for stepped-care approaches at these early times. However, we are only using self-submitted weight data (no doctor visit required), while the earlier work also included several baseline predictors.

As expected, the regression models (linear regression and support vector regression) showed worse prediction accuracy than the classification models. The regression models predict a weight change percentage, which is then converted into one of the classes according to the cutoff values, which is an additional error source. We did see this confirmed in our results, where at almost all time frames the prediction accuracy for the two regression models was trailing the prediction accuracy of the other models, with the exception of multi-layer perceptron. The lower prediction accuracy of multi-layer perceptron compared to the other classification models might be due to the training dataset being too small to properly train the neural networks.

The limitation of our models is that they rely on the reliability of the longitudinal data available from users. Especially in the beginning, with few weight entries for an individual, weight loss prediction might not be very reliable, until several entries of weight have been recorded. Additionally, the prediction might fail, if users change their behavior regarding the intervention, which cannot be predicted with this model.

In the future, this work could be improved in several ways. With more training and test data available for a longer time period, the model could be trained for prediction of weight loss after 12 months. Additional data sources could be explored as input to improve the prediction, for instance, when only few weight entries are available. Alternatively, additional prediction models other than the ones included in this work could be tested for their performance.

Overall, we showed that use of longitudinal weight data improved prediction accuracy for long-term weight loss prediction compared to previously published models only using baseline data. Additionally, we used a less error-prone data-source compared to energy-based prediction models, which should increase reliability of the prediction.

## Conclusion

We screened several machine learning models in their ability to predict weight loss in the HWC cohort and validated their performance using the independent dataset of the OxFAB study. We successfully implemented the best performing prediction model, logistic regression for both the three-class overall weight loss prediction and for the more refined five-class prediction.

The model is readily available to be used in a clinical setting, for instance, to help health professionals to automatically monitor individuals trying to lose weight and getting notified, if the individuals are not predicted to meet their weight loss goals.

The web application to demonstrate the use of the best-performing models is freely available: https://elolab.shinyapps.io/WeightChangePredictor/.

### Supplementary Information

Supplementary Figure S1.

Supplementary Information 2.

Supplementary Table S1.

Supplementary Table S2.

Supplementary Table S3.

Supplementary Information 6.

## Data Availability

Data described in the manuscript will not be made available because of compliance with the General Data Protection Regulation of the European Union. The code described in the manuscript will be made available upon reasonable request to the corresponding author.
