# Supplementary material for: Development and validation of a weight-loss predictor to assist weight loss management
Source: Sci Rep. 2023 Nov 24;13:20661. doi: 10.1038/s41598-023-47930-y (PMC10673897; doi:10.1038/s41598-023-47930-y)
Supplement: Supplementary file 6 — Supplementary Information 6. [file 41598_2023_47930_MOESM6_ESM.docx]

**Development and Validation of a Weight-Loss Predictor to Assist Weight Loss Management – Supplementary Text: Details of computational models**

This supplementary document provides additional details about the machine learning methods used for predicting the cluster-based nine-month weight-loss results as

$$y=f\left( x_{t} \right)+\epsilon_{t}$$

where $y$ is the observed weight loss outcome (either the five- or three-class weight loss result or the nine-month weight loss result in percentages), $f$ is an unknown function relating an input vector $x_{t}=\left( x_{t1},x_{t2},\ldots,x_{tp} \right)$ consisting of data available at time point $t$ to $y$, and $\epsilon_{t}$ is a random error term independent of $x_{t}$ and has mean zero and constant variance. To approximate $f$ and obtain predictions for $y$, we applied six supervised machine learning methods: logistic regression, linear regression, naive Bayes classification, support vector classification, support vector regression, and multi-layer perceptron. All models were implemented in *Python* (v 3.6.9) using the package *sklearn* (v 0.22.1) ^1^.

**Logistic regression**

Logistic regression model estimates the probability of an event based on a set of predictors ^2^. Here, to accommodate the prediction of multiple classes, we used the multinomial logit model ^3^, also known as softmax regression, where the probability for an input vector $x$ of belonging to cluster $c$ can be obtained from

$$P\left( y=c | x \right)=\frac{e^{\beta_{c0}+\beta_{c1}x_{1}+\ldots+\beta_{cp}x_{p}}}{\sum_{j=1}^{K} e^{\beta_{j0}+\beta_{j1}x_{1}+\ldots+\beta_{jp}x_{p}}}$$

where $\beta_{j0},\beta_{j1},\ldots,\beta_{jp}$ are the regression coefficients corresponding to each input variable for cluster $j$, and $K$ is the total number of clusters. In *sklearn*, the objective for optimization is

$$\min_{\beta}-\frac{1}{m}\sum_{i=1}^{p} \sum_{c=1}^{K} \left[ y=c \right]\log\left( P\left( y=c | x \right) \right)+r(\beta)$$

where $\left[ S \right]$ is the Iverson bracket returning 0 if $S$ is false and 1 otherwise, $m$ is the regularization strength, and $r(\beta)$ is the regularization term ^4^. By default, sklearn uses the L2 penalty and hence the model using the default settings is also known as ridge logistic regression. In this case, $r\left( \beta\right)=\frac{1}{2}\sum_{i=1}^{p} \sum_{j=1}^{K} \beta_{ij}^{2}$. Finally, among the set of all potential clusters $C$, the predicted cluster for each individual was the class with the highest probability

$$\hat{y}=arg \max_{c\in C} P\left( y=c | x \right).$$

For performing the logistic regression-based predictive modelling, we used the *LogisticRegression()* function with the following settings:

*linear_model.LogisticRegression(solver='saga', max_iter=1000, multi_class='multinomial')*

**Linear regression**

In the linear regression approach, we first estimated the percentage weight change at nine months as a linear combination of the predictors as

$$\hat{w}=\beta_{0}+\beta_{1}x_{1}+\ldots+\beta_{p}x_{p}$$

The regression coefficients were obtained using the ordinary least squares approach ^4^. Finally, to evaluate the classification accuracy, the estimated weight loss results were converted into the weight loss classes using cutoff values for weight change percentages as

$$\hat{y}=\left\{ c | r_{cl}\leq\hat{w}<r_{cu} \right\}$$

where $r_{cl}$ and $r_{cu}$ are the cluster-specific lower and upper cutoff values, respectively. The cutoff values were defined by sorting the observed weight change values after nine months of each individual from high to low and selecting the values minimizing the numbers of classification errors. If there were several values with the same error, we selected the largest cutoff value. The linear regression models were fit using the *linear_model.LinearRegression()* function with the default settings.

**Naïve Bayes classification**

The naïve Bayes classifier is a simple algorithm for predictive modelling based on the Bayes’ theorem ^5,6^. Here, to apply the method on continuous predictors, we used a Gaussian naïve Bayes classifier, a variant of the naïve Bayes technique in which the probability of an observed value belonging to class $c$ is computed by assuming a Gaussian distribution for the variable as

$$P\left( x_{i} | y=c \right)=\frac{1}{\sqrt{2\pi\sigma_{c}^{2}}}e^{\frac{-\left( x_{i}-\mu_{c} \right)^{2}}{2\sigma_{c}^{2}}}$$

where $\mu_{c}$ and $\sigma_{c}^{2}$ are mean and variance of values in input variable $x_{i}$ associated with class $c$ ^7^. To assign the predicted cluster labels, a Bayes classifier is applied as follows:

$$\hat{y}=\arg\max_{c\in C} P(c)\prod_{i=1}^{p} P\left( x_{i} | c \right)$$

The naïve Bayes classifiers were trained using the *naive_bayes.GaussianNB()* function with the default settings.

**Support vector classification**

Support vector machines are a set of supervised learning algorithms for classification and regression by finding an optimal hyperplane in a high-dimensional space that maximally separates different classes or output values ^8,9^. In a binary classification setting, given a set of input vectors $x$ with corresponding output classes $y$ coded as numerical values either -1 or 1, the goal is to learn a hyperplane with a normal vector $w=\left( w_{1},w_{2},\ldots,w_{p} \right)$ and offset parameter $b$ for which a prediction given by

$$\hat{y}={\mathrm{sign}(w}^{T}\phi\left( x \right)+b)$$

is correct for most samples ^10^. During this process, as an attempt to make the problem more linearly separable and consequentially improve performance, feature transform functions $\phi$ are applied to convert the input data into a higher-dimensional space. Conventionally, support vector machines do not support multi-class classification but can be rendered for the purpose by transforming the problem into multiple binary classification problems for each pair of classes and aggregating the results using a suitable decision function. Here, the support vector classification models were fit using the model instantiation *svm.SVC(kernel='linear')*, using default setting for all other parameters except the kernel type.

**Support vector regression**

With modifications in the numerical optimization procedure, support vector classification can be extended to support vector regression, suitable for solving regression problems and predicting a continuous response ^11^. Here, similar to linear regression, we used support vector regression to first estimate the percentage weight change at nine months and, finally, converted the estimated weight loss results into the weight loss classes using suitable cutoff values. The support vector regression models were fit using model instantation s*vm.SVR(kernel='linear').*

**Multilayer perceptron**

Multilayer perceptron is a feedforward artificial neural network, consisting of multiple fully connected layers that can learn a non-linear approximator suitable for classification or regression ^8,12^. The first layer in the network is the input layer containing data from the input variables. Following the input layer are the hidden layers that transform the values from the previous layer with a weighted linear summation of input values $w_{1}x_{1}+w_{2}x_{2}+\ldots+w_{p}x_{p}$ followed by a non-linear activation function such as the rectified linear unit (ReLU) defined as

$$\mathrm{ReLU}\left( z \right)=max(z,0)$$

The output layer receives the values from the last hidden layer and transforms them into output values according to pre-specified logic. Here, we used 5 hidden layers with ReLU activation for the 0.5 months model, then 5 additional hidden layers for each following model, up to 45 hidden layers at 8 months. To accommodate multi-class classification, softmax coding was used in the output function and, similarly as in logistic regression, the cluster with the highest probability was selected as the final output $\hat{y}$. As multi-layer perceptron model training is not deterministic, but includes randomness, all models were trained 500 times and the prediction accuracies of the 500 models were averaged for testing. The multilayer perceptron models were instantiated with the following call and settings (this example is given for the 0.5 months model):

*neural_network.MLPClassifier(hidden_layer_sizes=(5, ), solver='lbfgs', max_iter=1000)*

**References**

1. Pedregosa, F. et al. Scikit-learn: Machine Learning in Python. Journal of Machine Learning Research **12**, 2825–2830 (2011).

2. Le Cessie, S. & Van Houwelingen, J. C. Ridge Estimators in Logistic Regression. Journal of the Royal Statistical Society. Series C (Applied Statistics) **41**, 191–201 (1992).

3. Hausman, J. & McFadden, D. Specification Tests for the Multinomial Logit Model. Econometrica **52**, 1219–1240 (1984).

4. 1.1. Linear Models. scikit-learn https://scikit-learn/stable/modules/linear_model.html.

5. John, G. H. & Langley, P. Estimating continuous distributions in Bayesian classifiers. in Proceedings of the Eleventh conference on Uncertainty in artificial intelligence 338–345 (1995).

6. Zhang, H. The Optimality of Naive Bayes. in Proceedings of the Seventeenth International Florida Artificial Intelligence Research Society Conference, Miami Beach, Florida, USA (2004).

7. 1.9. Naive Bayes. scikit-learn https://scikit-learn/stable/modules/naive_bayes.html.

8. Sarker, I. H. Machine Learning: Algorithms, Real-World Applications and Research Directions. SN Computer Science **2**, 160 (2021).

9. Keerthi, S. S., Shevade, S. K., Bhattacharyya, C. & Murthy, K. R. K. Improvements to Platt’s SMO Algorithm for SVM Classifier Design. Neural Computation **13**, 637–649 (2001).

10. 1.4. Support Vector Machines. scikit-learn https://scikit-learn/stable/modules/svm.html.

11. Drucker, H., Burges, C. J. C., Kaufman, L., Smola, A. & Vapnik, V. Support Vector Regression Machines. in Advances in Neural Information Processing Systems vol. 9 (MIT Press, 1996).

12. 1.17. Neural network models (supervised). scikit-learn https://scikit-learn/stable/modules/neural_networks_supervised.html.
